# Effect of Altitude on Veteran Suicide Rates

**DOI:** 10.1089/ham.2018.0130

**Published:** 2019-06-21

**Authors:** Hana Sabic, Brent Kious, Danielle Boxer, Colleen Fitzgerald, Colin Riley, Lindsay Scholl, Erin McGlade, Deborah Yurgelun-Todd, Perry F. Renshaw, Douglas G. Kondo

**Affiliations:** ^1^ Brain Institute, University of Utah, Salt Lake City, Utah.; ^2^ Department of Psychiatry, University of Utah, Salt Lake City, Utah.; ^3^ Veterans Integrated Service Network 19 Mental Illness Research Education Clinical, Centers of Excellence, Salt Lake City Veterans Affairs Medical Center, Salt Lake City, Utah.

**Keywords:** altitude, elevation, suicide, veterans

## Abstract

***Aims:*** Suicide rates in the general population in the United States are correlated with altitude. To explore factors contributing to suicide among military veterans, we examined the relationship between veteran state-level suicide rates and altitude for 2014, including firearm-related and nonfirearm-related rates.

***Methods:*** Pearson's coefficients were calculated for altitude and each outcome. Mixed linear models were used to determine the association between suicide and altitude while adjusting for demographic confounds.

***Results:*** State mean altitude was significantly correlated with total veteran suicide rate (*r* = 0.678, *p* < 0.0001), veteran firearm-related suicide rate (*r* = 0.578, *p* < 0.0001), and veteran nonfirearm suicide rate (*r* = 0.609, *p* < 0.0001). In mixed models, altitude was significantly correlated with total veteran suicide rate (β = 0.331, *p* < 0.05), veteran firearm suicides (β = 0.282, *p* < 0.05), and veteran nonfirearm suicides (β = 0.393, *p* < 0.05).

***Conclusion:*** This study adds to evidence linking altitude and suicide rates, arguing for additional research into the relationship between altitude and suicide among veterans.

## Introduction

Suicide is a significant problem among military veterans in the United States (Kang et al., 2015). Suicide rates in the U.S. population as a whole have increased in the last decade (Curtin et al., 2016; Olfson et al., 2017), becoming one of the leading causes of death among young adults (Heron, 2018). The national veteran suicide rate has been reported to be more than two times that of the general population; it is estimated that of 100,000 veterans, 38.4 commit suicide per year, compared with 17.0/100,000 persons per year in the general population (U.S. Department of Veterans Affairs, 2016). Greater than expected increases have been seen in firearm-related suicides among veterans, with a 16.2% relative increase from 2001 to 2010. Among female veterans, even larger increases have been seen, with a 34.6% relative increase in overall suicide mortality rates and a 75.4% relative increase in firearm-related suicide rates from 2001 to 2010 (McCarten et al., 2015).

Such findings highlight the need to identify and understand factors that contribute to suicide risk among veterans and the general population (U.S. Department of Veterans Affairs, 2018). Suicide risk factors for veterans that have already been identified include psychiatric disorders such as major depression and other mood disorders (Joiner et al., 2005; Pfeiffer et al., 2009; Kaplan et al., 2012; Pompili et al., 2013; Lee et al., 2018), medical illness and associated disability (Kaplan et al., 2007), chronic pain and traumatic brain injury (Blakey et al., 2018), and substance abuse (Kimbrel et al., 2018; Lee et al., 2018). Likewise, combat exposure and the associated trauma are clearly linked to increased suicide risk for veterans (Thomas et al., 2017).

Firearm ownership is a risk factor for death by suicide in the general population (Miller et al., 2015; Siegel and Rothman, 2016), and veterans are more likely to own firearms: Cleveland et al. (2017) estimated, from a nationally representative sample of 1044 veterans, that 44.9% of veterans own a firearm, whereas only 22% of nonveterans in the United States own a firearm (Azrael et al., 2017). Together, these facts suggest that increased rates of firearm ownership among veterans may contribute to their increased suicide risk.

In addition to these commonly recognized suicide risk factors, however, multiple studies have shown that overall suicide rates are significantly correlated with altitude of residence. In the United States, state-level suicide rates have been shown to correlate with state peak altitude and capital city altitude (Haws et al., 2009), while suicide rates in capital counties are correlated with altitude (Cheng et al., 2005), and county-level suicide rates have been shown to correlate with both county center altitude (Brenner et al., 2011a) and mean county altitude (Kim et al., 2011) even after controlling for confounds such as population density and rates of firearm ownership. Most recently, Ha and Tu (2018) used geographically weighted regression models, while controlling for rates of firearm ownership, smoking, and other covariates, to demonstrate that U.S. county-level suicide rates are positively correlated with county mean altitude even after allowing for the possibility of regional variation in the strength of the relationship.

Associations between altitude and suicide rates have also been demonstrated in other countries, including Spain (Alameda-Palacios et al., 2015), South Korea (Kim et al., 2014a), and Austria (Helbich et al., 2013). Given these findings, we hypothesized that mean state altitude is positively associated with state-level suicide rates among veterans, even after controlling for possible confounds.

Conducting this analysis with veteran-specific data is important because of the high rates of suicide in veterans and because factors that contribute to suicide rates among veterans may differ from those among civilians (Martin et al., 2009; Hoffmire et al., 2015). Moreover, evidence that assesses the association between suicide and altitude in previously unstudied subpopulations has the potential to provide important confirmation or disconfirmation of the still relatively novel hypothesis that increased altitude is associated with higher suicide rates.

## Methods

### Data

U.S. Department of Veteran Affairs State Data Sheets were used to extract data related to age-adjusted state veteran suicide rates (calculated as the number of suicides per 100,000 veterans residing in the state) collected for the year 2014. Matching nonveteran suicide rates were also available. Veteran suicide rates for the age groups 18–34, 35–54, 55–74, and 75+ were also obtained (calculated as the number of suicides per 100,000 veterans in the relevant age group residing in the state per year). Shuttle Radar Topography Mission (SRTM) data, developed by the National Geospatial-Intelligence Agency, were used to calculate the mean altitude for each state. SRTM is a digital topographical global data set created in 2000 with a spatial resolution of 0.1 km. Digital altitude information was not available for Alaska and Hawaii; for that reason, data from these states were excluded, with 48 states remaining in the analysis.

### Independent variable identification

Upon evaluating the relationship between altitude and veteran suicide risk, we sought to control for confounding variables that might be correlated both with suicide risk and with altitude. State-level firearm ownership data were used as reported by Kalesan et al. (2016), who derived them from a nationally representative survey by YouGov (www.yougov.com) among individuals aged >18 years in the United States in 2013. No veteran-specific information regarding rates of firearm ownership by state could be identified. As cigarette smoking is associated with suicide risk (Balbuena and Tempier, 2015; Ha and Tu, 2018) and might be influenced by altitude or location, state-level smoking rates were obtained from the Centers for Disease Control and Prevention State Tobacco Activities Tracking and Evaluation (STATE) System (Centers for Disease Control and Prevention, 2018). Again, no veteran-specific state-level data regarding smoking prevalence could be identified.

Veteran-specific covariate data were obtained from the Department of Veterans Affairs (2018). We acquired the following state-level variables, each separately representing both rural and urban veteran populations in each state: percentage veteran population; percentage female veteran population; veteran unemployment rate; veteran poverty rate; veteran disability rate; percentage of veterans with no high school degree; percentage of veterans with some college; percentage of veterans with bachelor's degree or greater; number of Department of Veterans Affairs (VA) facilities in the state; and use of VA services. Each of these factors was regarded as potentially correlating with both altitude and suicide risk.

We considered including urban- and rural-specific versions of the above variables (e.g., disability rate among urban veterans and disability rate among rural veterans) because of the established associations between population density and suicide (Frankel, 1992; Hempstead, 2006; Hirsch, 2006; Murphy, 2014; Helbich et al., 2017) and between population density and higher altitude (Cohen and Small, 1998). Because the available data were sparse, however, including figures for only 48 states, we endeavored to minimize the risk of overfitting by combining similar variables (Babyak, 2004), in this case rural and urban variables (e.g., rural veteran disability rate and urban veteran disability rate), by taking the averages of these figures weighted for the rural and urban distributions of veteran populations in each state. Similarly, we included only the percentage of veterans with no high school degree in our analysis, as the percentages of veterans with high school degrees or college degrees should be collinear with this. After excluding combined variables that were not significantly correlated with suicide rates in univariate analyses, six variables were included in our multivariable analyses.

### Statistical analysis

Statistical analyses were conducted with version 9.4 of the SAS System for Windows (Copyright © 2016, SAS Institute, Inc., Cary, NC). The associations between mean state altitude, total veteran suicide rates, veteran firearm suicide rates, and veteran nonfirearm suicide rates were assayed by calculating Pearson's correlation coefficients after visually verifying that the distributions of the outcomes were approximately normal. Likewise, we calculated Pearson's coefficients for state-level smoking and firearm ownership rates and mean state altitude. We assessed for spatial autocorrelation of suicide rates by first using the coordinates of the geographic centroids of each state (U.S. Geological Survey, 1964) to determine easting and northing distances from the most south-westerly point in our grid (the geographic centroid of Hawaii) using the Haversine formula (Robusto, 1957). Then, we computed Moran's I (Ord, 2010) for each outcome variable using the VARIOGRAM procedure in SAS. This demonstrated that total veteran suicide rates, veteran firearm suicide rates, and veteran nonfirearm suicide rates were significantly spatially autocorrelated (*p* < 0.0001 in each case).

To further analyze the association between veteran suicide rates and altitude while accounting for spatial autocorrelation, a mixed linear regression model for the variance in veteran suicide rates was constructed (with the MIXED procedure) with selected independent variables using restricted maximum-likelihood estimation and assuming a spatial Gaussian covariance structure using the same spatial grid defined above (Dormann et al., 2007). Covariates were included in the model if and only if they had been found to be associated with suicide rates in univariate testing. For each model, normalized regression coefficients for each model parameter and standard errors were calculated. Statistical significance for each parameter was defined as alpha <0.05 and two tailed. The same approach was used for our secondary analyses, in which we examined the association between rates of firearm suicides and nonfirearm suicides among veterans.

## Results

### Characteristics of the sample

Mean altitude data were available for 48 states (Table 1). The mean state altitude was 1717.2 ± 1853.5 ft, with the lowest mean altitude being 58.8 ft (Delaware) and the highest being 7192.2 ft (Colorado). The mean suicide rate among veterans in all age groups was 41.5 ± 10.7 suicides per 100,000 per year; the highest state suicide rate was 68.6 (Montana), whereas the lowest was 21.6 (Massachusetts). Among states with altitude and suicide data, the mean population rate of firearm ownership was reported to be 32.2% ± 13.0%, with the highest rate in Arkansas (57.9%) and the lowest in Delaware (5.2%). Similarly, in this subset, the mean state smoking rate was 18.6% ± 3.48%, whereas the highest state smoking rate was 26.7% (West Virginia) and the lowest 9.7% (Utah).

**Table 1.** Distributions of Altitude, Suicide Rates, Smoking Rates, and Firearm Ownership Rates

*Variable*	*Mean*	*SD*	*Minimum*	*Maximum*
State mean altitude (ft)	1717.2	1853.5	58.8	7192.2
State veteran suicide rate (suicides/100,000 veterans/year)	41.5	10.7	21.6	68.6
State overall suicide rate (suicides/100,000 persons/year)	19.2	4.68	10.6	30.2
State veteran nonfirearm suicide rate (suicides/100,000 veterans/year)	13.5	4.09	6.14	24.9
State veteran firearm suicide rate (suicides/100,000 veterans/year)	27.8	8.19	8.57	43.8
Overall nonfirearm suicide rate (suicides/100,000 persons/year)	6.9	1.45	4.24	9.95
Overall firearm suicide rate (suicides/100,000 persons/year)	7.85	2.85	1.9	14
Smokers in state (%)	18.6	3.48	9.7	26.7
Firearm owners in state (%)	32.2	13	5.2	57.9

### Associations among covariates

To assess for relationships among our major confounds, we calculated Pearson coefficients for the correlations between state mean altitude and rates of smoking and firearm ownership. State smoking rates were significantly negatively correlated with altitude (*r* = −0.311, *p* < 0.05), while firearm ownership rates were significantly positively correlated with altitude (*r* = 0.366, *p* < 0.05).

### Correlations between veteran suicide rates and altitude

**FIG. 1.**
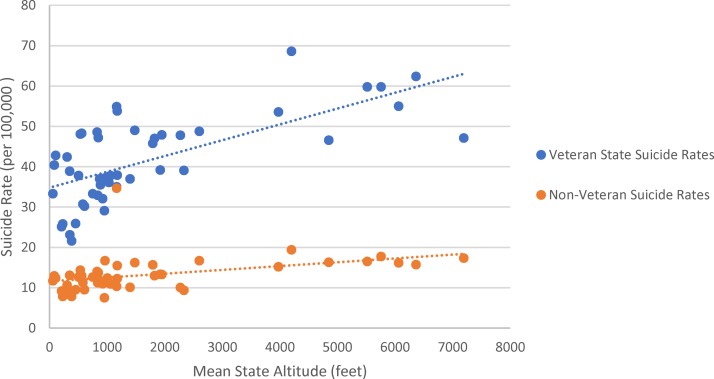
Scatterplot of veteran and nonveteran suicide rates and mean altitude in the contiguous U.S. states.

**FIG. 2.**
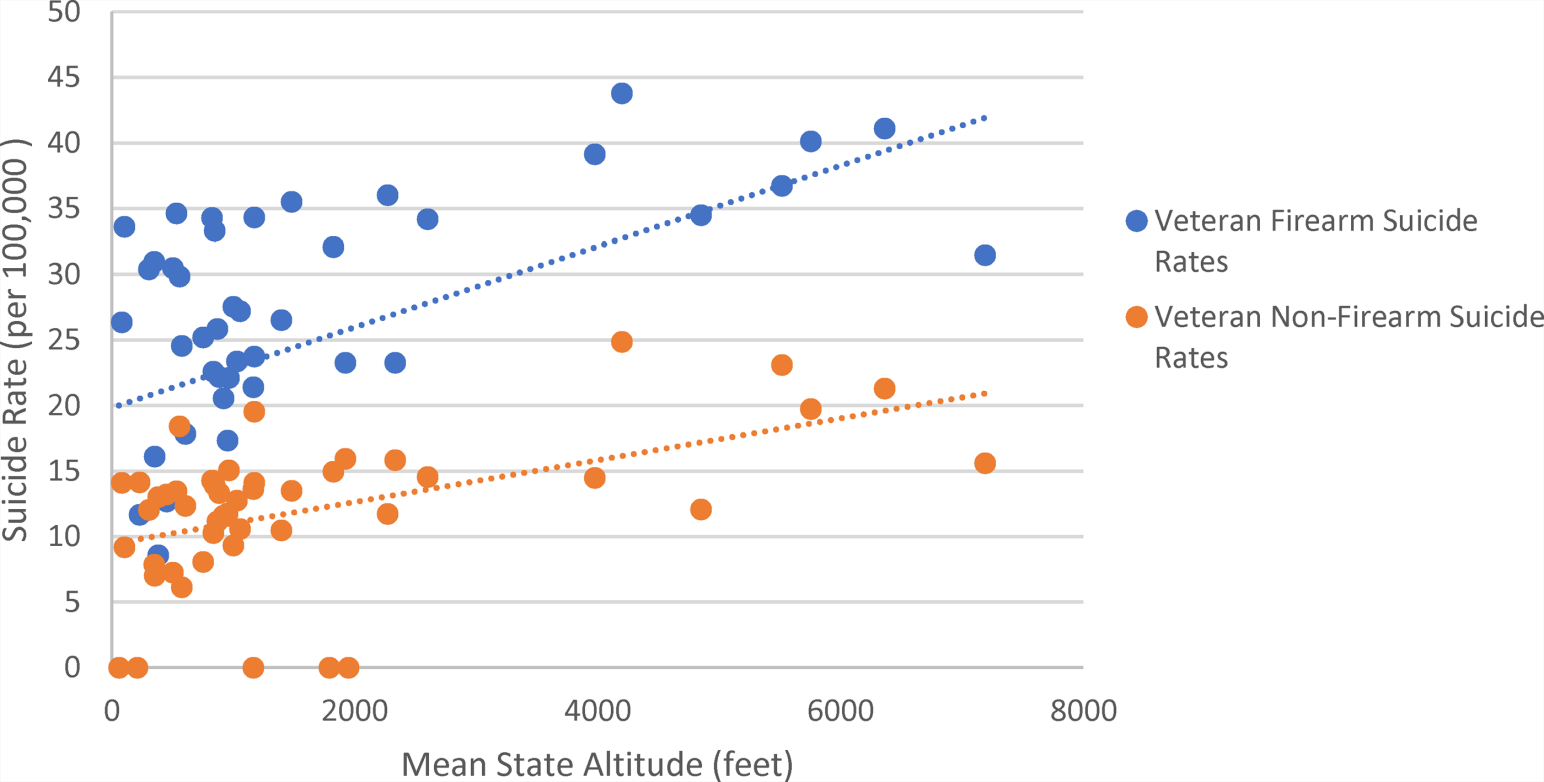
Scatterplot of veteran firearm and nonfirearm suicide rates and mean altitude in the contiguous U.S. states.

As shown in Table 2 and Figure 1, there is a significant positive correlation between veteran suicide rates and state mean altitude in the United States. A stronger correlation between suicide rate and altitude was found in veterans (*r* = 0.678, *p* < 0.0001) than in nonveterans (*r* = 0.409, *p* = 0.004). Age group data were lacking for some states, as noted. State mean altitude was significantly correlated with both veteran firearm-related suicide rate (*r* = 0.578, *p* < 0.0001) and veteran nonfirearm suicide rate (*r* = 0.609, *p* < 0.0001) (Fig. 2).

**Table 2.** Correlations Between Altitude and Veteran Suicide Rates by Age Group and Nonveterans in U.S. States

*Suicide rate*	*State mean altitude*	
	r	p
Nonveterans	0.409^a^	0.004
Veterans	0.678^a^	<0.0001
Veteran nonfirearm	0.609^b^	<0.0001
Veteran firearm	0.578^b^	<0.0001
Veterans 18–34	0.125^c^	0.473
Veterans 35–54	0.475^d^	0.001
Veterans 55–74	0.579^e^	<0.0001
Veterans 75+	0.737^f^	<0.0001

States with <10 suicides per age group were excluded from the analysis.

^a^ Based on 48 states (excluding AK and HI).

^b^ Based on 42 states (excluding AK, DE, HI, ND, RI, SD, VT, and WY).

^c^ Based on 35 states (excluding AK, CT, DE, HI, ID, IO, ME, MT, NH, ND, RI, SD, VT, WV, and WY).

^d^ Based on 45 states (excluding AK, DE, HI, RI, and WY).

^e^ Based on 41 states (excluding AK, DE, HI, ID, ND, RI, SD, VT, and WY).

^f^ Based on 36 states (excluding AK, CT, DE, HI, IO, ME, MT, NH, ND, RI, SD, VT, WV, and WY).

In addition, our results suggest that veteran firearm suicide rates are greater than veteran nonfirearm suicide rates. These findings support previous research, indicating that 66% of veteran suicides in 2014 were related to firearm injuries (Department of Veterans Affairs Suicide Prevention Program, 2016). It is noteworthy, however, that both Figures 1 and 2 indicate that other factors must also contribute to suicide risk, as the state with the highest average altitude (Colorado) had lower total and firearm-related veteran suicide rates than some other, lower altitude states.

Table 2 shows that the correlation between veteran suicide and altitude appears to begin in middle age, and to increase across the lifespan; the correlation between altitude and suicide in the youngest quartile of veterans (ages 18–34) was not significant, but became significant in older groups, with the greatest correlation in veterans who are >75 years (*r* = 0.737, *p* < 0.0001).

### Multivariable analysis

To explore these results further, we conducted multiple regression analyses, assuming a spatial Gaussian covariance structure, including available covariates that exhibited significant associations with suicide rates in univariate testing. Table 3 presents the standardized correlation coefficients from multiple regression analyses for the total veteran suicide rate, veteran firearm suicide rate, and veteran nonfirearm suicide rate. Mean state altitude was significantly associated with total veteran suicide rate (*β* = 0.331, *p* < 0.05). Other factors included in the model, though significantly associated with total veteran suicide rates in univariate analyses, were no longer so in the multivariable analysis.

**Table 3.** Standardized Correlation Coefficients (*β* Coefficients) in U.S. States for Total Veteran Suicide Rates, Veteran Nonfirearm Suicide Rates, and Veteran Firearm Suicide Rates

*Measure*	*Total veteran suicides*	*Veteran nonfirearm suicides*	*Veteran firearm suicides*
Mean state altitude	0.331^a^	0.393^a^	0.282^a^
Firearm ownership rate	NS	NS	NS
Veteran unemployment rate	NS	NS	NS
Veteran poverty rate	NS	NS	NS
Veteran disability rate	NS	NS	NS
Veterans with no high school diploma	NS	NS	NS

Only significant values shown; ^a^*p* < 0.05.

NS, nonsignificant.

Multiple regressions were also performed for veteran nonfirearm suicide rates and veteran firearm suicide rates. The analyses indicated that state mean altitude is significantly correlated with veteran nonfirearm suicides (*β* = 0.393, *p* < 0.05), though other included independent variables were not. Veteran firearm suicide rates were significantly associated with both state mean altitude (*β* = 0.282, *p* < 0.05) and state firearm ownership rates (*β* = 0.318, *p* < 0.05), but were not significantly associated with other independent variables included in the model.

## Discussion

This study, to the best of our knowledge, is the first to suggest that U.S. military veterans living at higher altitudes are at increased risk of suicide. Our findings are in agreement with prior studies, which indicated that altitude is associated with suicide in the general population (Joiner et al., 2005; Brenner et al., 2011a; DelMastro et al., 2011; Gamboa et al., 2011). The association between total veteran suicide rates and altitude remained even after controlling for available confounding variables. When examining veteran firearm as well as nonfirearm suicide rates, mean state altitude continued to be significantly associated with each.

Although our results indicate that altitude of residence may serve as an important predictor of veteran suicide, we found that other geographic factors may also contribute. In univariate analyses, total veteran suicide rates, veteran nonfirearm suicide rates, and veteran firearm suicide rates were significantly correlated with veteran disability, poverty, unemployment, and education, although these associations were not replicated in multivariable analyses after accounting for the spatial autocorrelation of suicide rates. We also found that veteran firearm suicides were significantly associated with state firearm ownership rates, even in our multivariable analysis.

Although our findings do not show that higher altitude contributes causally to suicide risk among veterans, they invite speculation about mechanisms that might connect altitude and suicide. Although the slight decrease in arterial oxygen concentration associated with living chronically at moderate elevation has not been shown to have clinical effects, it has been suggested that residing at higher altitudes may result in chronic relative hypoxia, which could in turn impact brain processes such as monoamine synthesis and bioenergetics (Brenner et al., 2011b; Fiedler et al., 2012; Kim et al., 2014b; Kious et al., 2018).

A plausible link exists between decreased serotonin synthesis, which is oxygen dependent (Davis et al., 1973), and increased altitude. The arterial partial pressure of oxygen in humans decreases not only at extreme altitude (Grocott et al., 2009) but also at moderate altitudes (between 1400 and 3350 m) (Crapo et al., 1999; Gahutu et al., 2005; Pereira-Victorio et al., 2014; Sayeed et al., 2017). The contribution of smoking to relative hypoxia may be one reason smoking is often positively correlated with suicide risk. Still, no studies to date have demonstrated in humans that exposure to altitude is directly associated with reduced monoamine neurotransmitter synthesis; although studies in animals have found such an association (Ray et al., 2011; Bogdanova et al., 2014), this was observed primarily at much higher simulated altitudes (e.g., >3000 m).

Although these findings indicate an association between mean state altitude and veteran suicide rates, they should be interpreted carefully, as our study has several limitations. Due to limited access to veteran-specific data, we were unable to incorporate several variables into the analyses, including demographic factors, veteran medical and psychiatric histories, and cultural components that may also affect suicide risk. We included total state smoking rates and firearm ownership rates, which also encompass nonveterans, but these figures may not accurately capture veteran-specific rates of smoking and firearm ownership.

Similarly, state mean altitude is a relatively coarse-grained measure of the altitude to which veterans in a state might be exposed; lack of access to substate-level veteran data, however, precluded a more fine-grained analysis. Still, we hope that this study will serve as a basis for further work in this domain using more detailed data.

Connected to this limitation is that it is impossible, with currently available data, to address the modifiable areal unit problem; that is, the problem that the strength of the association between an independent variable and a geographically distributed outcome can vary depending on the size of the geographic area assumed in the analysis (Fotheringham and Wong, 1991). Again, this limitation might be addressed through future work. Moreover, the data available do not represent the duration of exposure to a given altitude. Further investigation will be needed to understand the association between altitude and rates of suicide in veterans.

Our study included several unexpected findings, some of which suggest areas for further study. We found (Fig. 1) that the association between suicide rate and altitude is stronger in veterans (*r* = 0.678, *p* < 0.001) than in nonveterans (*r* = 0.409, *p* = 0.004); should our findings be replicated, there will be an urgent need for research aimed at determining the reason(s) for this. Second, we found that the correlation between suicide rates and altitude was greater for older veterans. The explanation for this finding is unclear, but it raises the possibility that reduced physiologic reserves in older veterans might make them more vulnerable to the effects of altitude; it may also be due simply to age-dependent increases in veteran suicide rates. In any case, this phenomenon is one for VA clinicians to be mindful of and is worthy of further investigation.

## Conclusions

These results suggest that residence at higher altitudes may increase suicide risk among U.S. military veterans. Additional epidemiological research controlling for other confounding factors and using a more sensitive measure of geographic location, that is, altitude exposure among veterans, appears warranted, as do studies that examine mechanisms that might underpin the association.
